# The Contribution of TRPA1 to Corneal Thermosensitivity and Blink Regulation in Young and Aged Mice

**DOI:** 10.3390/ijms241612620

**Published:** 2023-08-09

**Authors:** Laura Frutos-Rincón, Carolina Luna, Fernando Aleixandre-Carrera, Enrique Velasco, Ariadna Diaz-Tahoces, Víctor Meseguer, Juana Gallar, M. Carmen Acosta

**Affiliations:** 1 Instituto de Neurociencias, Universidad Miguel Hernández-Consejo Superior de Investigaciones Científicas, 03550 San Juan de Alicante, Spain; l.frutos@umh.es (L.F.-R.); carolina.luna@umh.es (C.L.); f.aleixandre@umh.es (F.A.-C.); e.velasco@umh.es (E.V.); adiaz@umh.es (A.D.-T.); vmeseguer@umh.es (V.M.); juana.gallar@umh.es (J.G.); 2 Instituto de Investigación Biomédica y Sanitaria de Alicante, 03010 Alicante, Spain

**Keywords:** TRPA1, thermosensitivity, cold thermoreceptor, aging, corneal nerves, tearing, blinking

## Abstract

The role of TRPA1 in the thermosensitivity of the corneal cold thermoreceptor nerve endings was studied in young and aged mice. The contribution of the TRPA1-dependent activity to basal tearing and thermally-evoked blink was also explored. The corneal cold thermoreceptors’ activity was recorded extracellularly in young (5-month-old) and aged (18-month-old) C57BL/6WT (WT) and TRPA1^−/−^ knockout (TRPA1-KO) mice at basal temperature (34 °C) and during cooling (15 °C) and heating (45 °C) ramps. The blink response to cold and heat stimulation of the ocular surface and the basal tearing rate were also measured in young animals using orbicularis oculi muscle electromyography (OOemg) and phenol red threads, respectively. The background activity at 34 °C and the cooling- and heating-evoked responses of the cold thermoreceptors were similar in WT and TRPA1-KO animals, no matter the age. Similar to the aged WT mice, in the young and aged TRPA1-KO mice, most of the cold thermoreceptors presented low frequency background activity, a low cooling threshold, and a sluggish response to heating. The amplitude and duration of the OOemg signals correlated with the magnitude of the induced thermal change in the WT but not in the TRPA1-KO mice. The basal tearing was similar in the TRPA1-KO and WT mice. The electrophysiological data suggest that the TRPA1-dependent nerve activity, which declines with age, contributes to detecting the warming of the ocular surface and also to integrating the thermally-evoked reflex blink.

## 1. Introduction

The Transient Receptor Potential Ankyrin 1 (TRPA1) channel is a polymodal detector involved in thermal, mechanical, and chemical nociception. This nociceptive channel is expressed in the sensory neurons of the dorsal root ganglia, nodose ganglia, and trigeminal ganglia (TG) [1]. TRPA1 has been traditionally suggested as a noxious cold detector [2,3,4,5]. Although its cold sensing ability still remains controversial [6,7,8], growing evidence suggests that TRPA1 also plays a role in mild and noxious heat sensing [9,10,11,12]. On the other hand, TRPA1 is a sensor of a wide variety of exogenous and endogenous molecules, whose activation is involved in acute and chronic pain and inflammation (reviewed by Talavera et al. [13]).

The cornea is densely innervated by sensory unmyelinated (C) and thin myelinated (Aδ) axons [14,15,16] having their origin in the ophthalmic area of the TG [17] and ending centrally in two lower regions of the trigeminal brainstem complex: the trigeminal interpolaris/caudalis (Vi/Vc) transition and the Vc/upper cervical cord (Vc/C1) region [18,19,20,21]. Electrophysiological recordings of sensory nerve fibers innervating the cornea have revealed the existence of different functional types of ocular sensory neurons, classically classified based on the modality of stimulus by which they are preferentially activated [22]: polymodal nociceptors (around a 70% of the total) responding to mechanical forces, heat, and a wide variety of chemicals; mechanonociceptors (10–20%) responding to mechanical forces; and cold thermoreceptors (10%), which respond to moderate cooling, hyperosmolarity, and in some cases also to heating (the so-called paradoxical response to heat of the cold thermoreceptor neurons) [16,23,24,25,26,27]. In addition to evoking thermal sensations, sensory input about ocular surface temperature changes plays an important role in the regulation of protective mechanisms such as tearing and blinking. The specific sensitivity of corneal sensory neurons to each type of stimulus is determined by the expression of distinct transducing channels, including several members of the Transient Receptor Potential family (TRPs) [28] whose activity plays a crucial role in several regulatory processes and protective mechanisms of the ocular surface, such as the aforementioned tearing and blinking [29,30,31,32,33]. Indeed, the Transient Receptor Potential cation channel subfamily M member 8 (TRPM8) is specifically gated by cold [34,35], and its deletion in mice eliminates cold-evoked activity and reduces basal tearing [31]. TRPM8 has been proposed also as an osmosensor responsible for the regulation of basal blinking in mice [26,33], as its deletion or blockade also lead to a reduced blink frequency. The contribution of TRPA1 to the activity of the cold sensory nerve endings has not been established yet. The activation of TRPA1 and TRPV1 seems to contribute to eye discomfort sensations in allergic keratoconjunctivitis, given that their pharmacological blockade prior to the allergic challenge prevented the significant blinking and tearing rate caused by exposure to the allergen [36]. Furthermore, it has been proposed that TRPA1-related mechanisms are pivotal in mediating the sensitizing effects of chronic tear deficiency on trigeminal brainstem neuron excitability, as persistent tear reduction enhances the nocifensive behavior and trigeminal brainstem activity evoked by TRPA1 agonists [37].

Sensitivity to innocuous warm and cold stimuli tends to decline gradually with age in the skin and cornea of humans [38,39], although the basis of this decline in thermosensitivity remains unclear due to the scarce animal studies about the changes in sensory nerve activity and the implication of TRP channels to the reduced thermal sensitivity. The changes in the expression and functionality of TRPM8 of trigeminal cold thermosensitive neurons in aged (24-month-old) TRPM8-EYFP mice have been described. In aged animals, most of the TRPM8+ trigeminal neurons and their axons innervating the cornea (about 90%) were weakly fluorescent (revealing a low expression of TRPM8) and exhibited very low background impulse activity and an abnormal responsiveness to cooling pulses [40]. On the contrary, no reports have been published until now about the changes in the TRPA1 with age.

In the present work, we investigated the contribution of TRPA1 to corneal cold thermoreceptors’ thermosensitivity and the possible contribution of their TRPA1-dependent activity to the regulation by the CNS of basal tearing and the thermally-evoked blink reflex. For this purpose, ex vivo electrophysiological recording of corneal nerve terminals, basal tearing volume measurement, and orbicularis oculi muscle electromyography (OOemg) recording were performed in C57BL/6WT (WT) and TRPA1 knockout (TRPA1-KO) mice. The data obtained in basal conditions and after application of different thermal stimuli to the ocular surface were compared. A set of experiments were conducted in aged WT and TRPA1-KO mice in order to define the changes with aging.

## 2. Results

### 2.1. Corneal Cold Thermoreceptors’ Activity in WT and TRPA1-KO Young Mice

To study the effects of TRPA1 channel deletion in corneal thermosensitivity, the activity of the corneal cold thermoreceptor nerve terminals in response to thermal stimulation was recorded ex vivo in the eyes of WT and TRPA1-KO young mice (see Methods, Section 4.2).

#### 2.1.1. Background Activity and Cold-Evoked Responses

The background impulse activity of corneal cold thermoreceptors at a constant temperature of 34 °C and their response to cooling ramps from 34 °C to 15 °C were studied.

**Figure 1:**
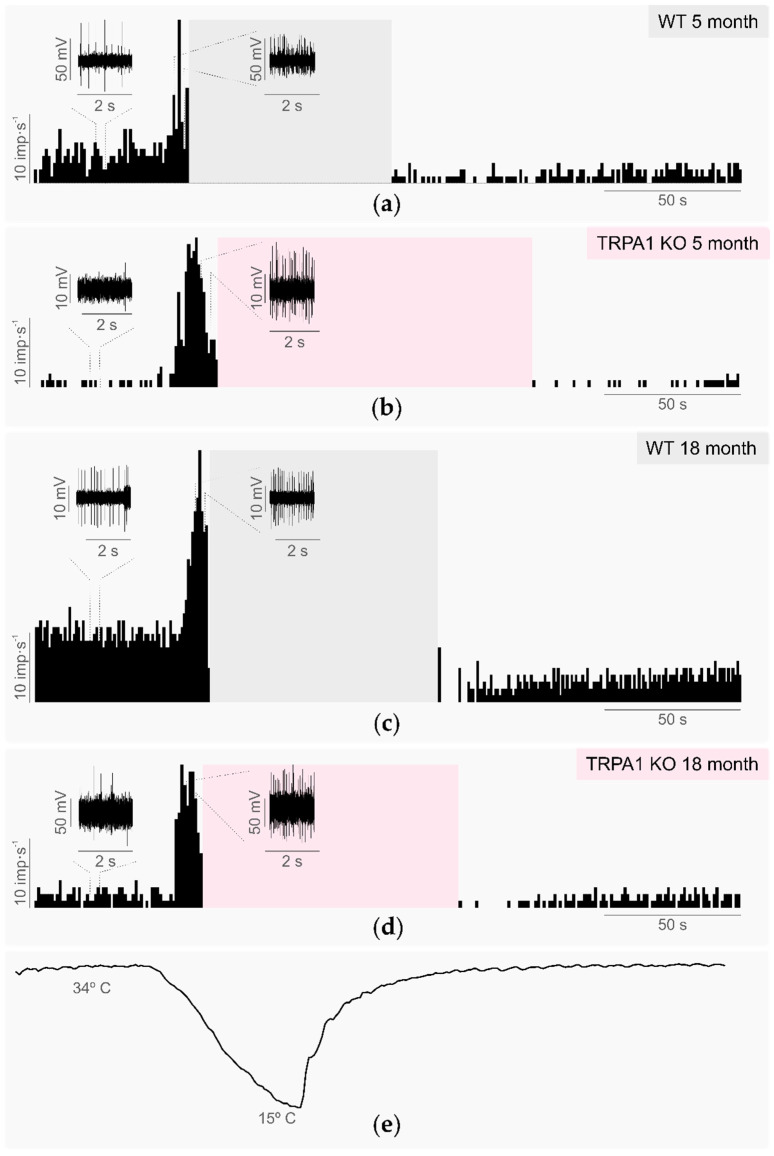
Response to the cooling of cold nerve terminals recorded in young and aged wildtype and TRPA1-KO mice. (**a**–**d**) Histograms showing the discharge rate in imp.s^−1^ of a single cold nerve terminal representative of each experimental group: (**a**) WT 5-month-old mice; (**b**) TRPA1-KO 5-month-old mice; (**c**) WT 18-month-old mice; (**d**) TRPA1-KO 18-month-old mice. The insets show the nerve terminal impulses recorded at the basal temperature of 34 °C (background activity) and during the maximal response to the cooling ramp (peak frequency). The shaded areas indicate the time to recover nerve firing (return time). (**e**) The temperature of the solution inside the recording chamber.

The background activity was not significantly different in young TRPA1-KO compared with age-matched WT mice (Table 1, Figure 1), which suggests that TRPA1 is not essential for determining cold thermoreceptor background firing at the basal temperature of the cornea. Similarly, the different parameters analyzed to characterize the response to cooling ramps (cooling threshold, cooling response, peak frequency, temperature to reach the peak frequency, and silencing temperature during the cooling ramp; see Methods, Section 4.2.2.1) were not significantly different in young WT and TRPA1-KO animals, with an average temperature reduction of 2.2–2.4 °C needed to reach the cooling threshold and evoke an increase in the firing activity (Table 1, Figure 1). The cold-evoked firing response reached its maximum value (peak frequency) when the temperature was reduced by 4.5–5 °C. The firing rate decreased gradually afterwards and completely disappeared at temperatures 6.5–7.1 degrees below basal (Table 1, Figure 1). Of note, as the corneal cold thermoreceptor activity almost completely disappeared at temperatures below 27 °C in both the WT and TRPA1-KO young animals (see silencing temperature during cooling ramp; Table 1, Figure 1), the proposed role of TRPA1 in the response to temperatures between 27 °C and 15 °C cannot be ruled out.

**Figure 2:**
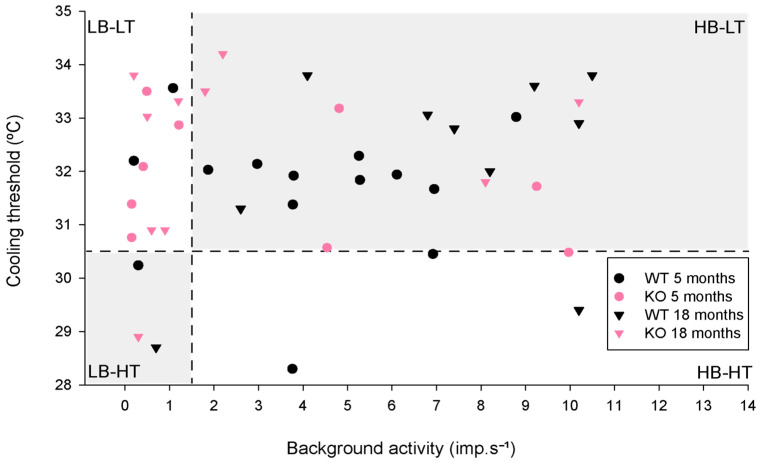
Distribution of the corneal cold thermoreceptors recorded in young (5-month-old) and aged (18-month-old) wildtype (WT) and TRPA1 knockout (KO) mice. Cold thermoreceptors were considered high background (HB) when their mean background activity at 34 °C was equal or over 1.5 imp.s^−1^ (vertical dashed line); otherwise, they were considered low background (LB). Cold thermoreceptors were considered high threshold (HT) when their cooling threshold, that is, the temperature needed to evoke the response to a cooling ramp from 34 °C to 15 °C, was equal to or below 30.5 °C (horizontal dashed line). WT 5 months, n = 14; KO 5 months n = 9; WT 18 months n = 10; KO 18 months, n = 10.

Based on their background activity at basal temperature and their threshold to cooling stimulation, corneal cold thermoreceptors can be classified as high background–low threshold (HB-LT) and low background–high threshold cold thermoreceptors (LB-HT) [16,27] (see Methods, Section 4.2.2.1), with intermediate behaviors (high background–high threshold, HB-HT; low background–low threshold, LB-LT) found in aged animals and in injured corneas [40,41]. In the present work, the TRPA1-KO mice presented a fraction higher, but not statistically significant, LB-LT cold thermoreceptors than the WT mice (55.6% TRPA1-KO vs. 14.3% WT, *p* = 0.102, z-test; Figure 2, pink and black circles, respectively).

#### 2.1.2. Sensitivity to Warm and Heat Stimuli

In WT corneas, the cold thermoreceptor activity was silenced when temperatures below 27 °C were reached during the cooling ramp from 34 °C to 15 °C. When warming from 15 °C to 34 °C after the cooling ramp, impulse activity reappeared and eventually recovered the basal values. The silencing at low temperatures and the recovery of impulse activity during warming back to 34 °C were similar in the TRPA1-KO mice, although the temperature and time needed to recover the impulse activity was significantly higher in the TRPA1-KO than in the WT animals (Table 1, Figure 1).

**Figure 3:**
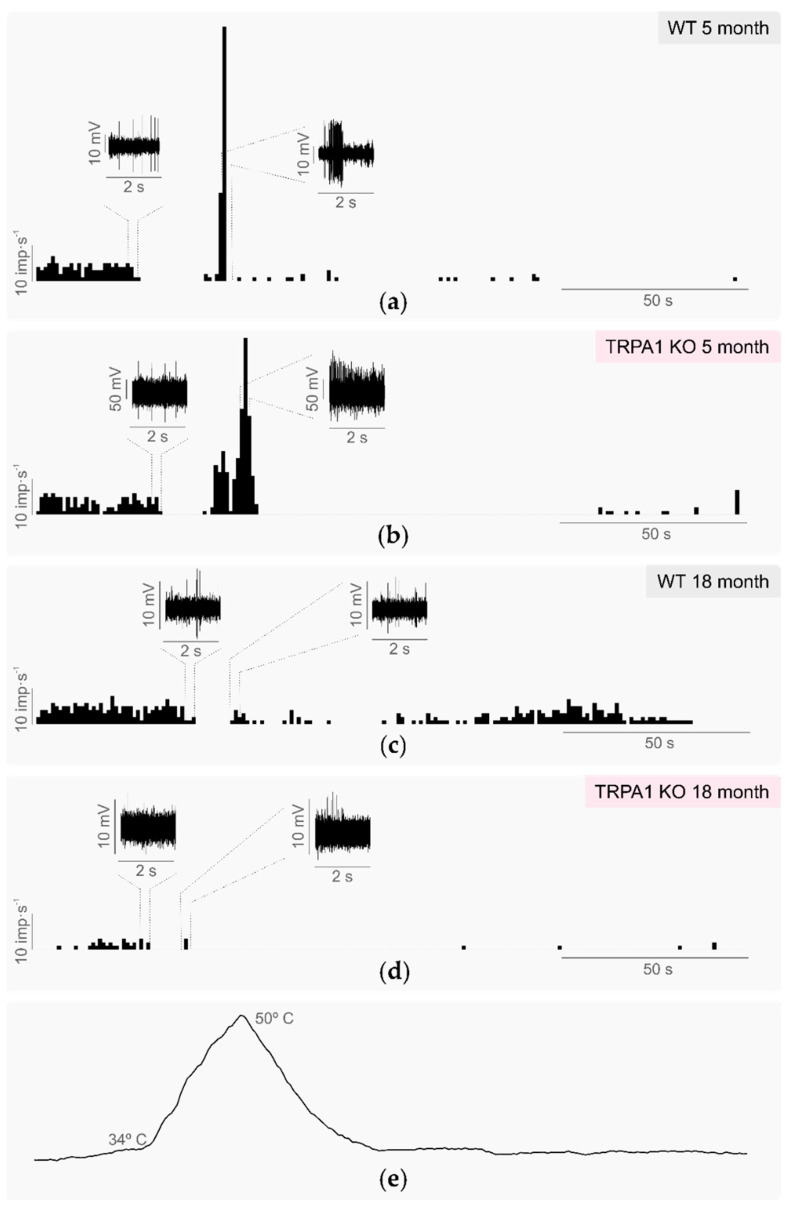
Response to heating of the cold nerve terminals recorded in young and aged wildtype and TRPA1-KO mice. (**a**–**d**) Histograms showing the discharge rate in imp.s^−1^ of a single cold nerve terminal representative of each experimental group: (**a**) WT 5-month-old mice; (**b**) TRPA1-KO 5-month-old mice; (**c**) WT 18-month-old mice; (**d**) TRPA1-KO 18-month-old mice. The insets show the nerve terminal impulses recorded just before being silenced and during the maximal response to heat. (**e**) The temperature of the solution inside the recording chamber.

During the heating ramp from 34 °C to 45 °C, all cold thermoreceptors decreased their firing rate, and about half of them were fully silenced at temperatures in the innocuous range (35–40 °C). This silencing temperature was slightly higher in the TRPA1-KO than in the WT mice (Figure 3), although the difference did not reach statistical significance (Table 1). At higher temperatures during the heating ramp (41–43 °C), most of the cold thermoreceptor terminals of both the WT and TRPA1-KO mice started again to fire (Figure 3), with the mean discharge rate during the heating ramp (paradoxical response to heat) twofold to threefold the value of the background activity at 34 °C (Table 1). As previously reported [11], the heating threshold temperature was slightly higher in the TRPA1-KO than in the WT mice (Table 1). Taken together, these results suggest that the absence of TRPA1 does not abolish the response of corneal cold thermoreceptors to noxious heating, although TRPA1 seems to be involved in recovery of firing activity during innocuous warming.

#### 2.1.3. Shape of the Nerve Terminal Impulses

Pioneering work on the mechanisms underlying the ion currents responsible for the different phases of the electrical impulses recorded with the focal recording technique showed that the variations over time of the shape of nerve terminal impulses (NTIs) of the cold thermoreceptor terminals at basal temperature, reflected the activity of the K^+^ channels [42]. On the other hand, some K^+^ channels interact functionally with TRPA1 and modulate TRPA1-mediated nociception in sensory neurons [43]. That led us to study the shape of the NTIs at basal temperature. In this condition, the NTIs were always biphasic in both the TRPA1-KO and WT nerve terminals (see Methods). The different parameters analyzed to define the NTI shape (+dV/dt, −dV/ms, Ratio, Width, T1 and T2, see Methods Section 4.2.2.2.) had similar values in the WT and TRPA1-KO nerve terminals (Table 2), which suggests that the TRPA1 does not contribute significantly to the shape of NTIs at a basal temperature of 34 °C.

### 2.2. Tearing and Blinking in Young WT and TRPA1-KO Mice

To define the contribution of TRPA1-mediated activity to the physiological functions depending on cold thermoreceptor activity, the basal tearing and the thermally evoked blinking were studied in 5-month-old WT and TRPA1-KO mice (see Methods, Section 4.3 and Section 4.4).

#### 2.2.1. Basal Tearing Volume

No differences were found in the basal tearing volume measured in the WT and TRPA1-KO animals (1.3 ± 0.3 vs. 1.5 ± 0.4 mm, WT vs. TRPA1-KO, n = 10 and 8 eyes, respectively; *p* = 0.735, *t*-test), which suggests that TRPA1-dependent activity is not necessary to maintain the basal tearing values at physiological conditions.

#### 2.2.2. Blinking Evoked by Cooling and Heating Stimulation of the Ocular Surface

**Figure 4:**
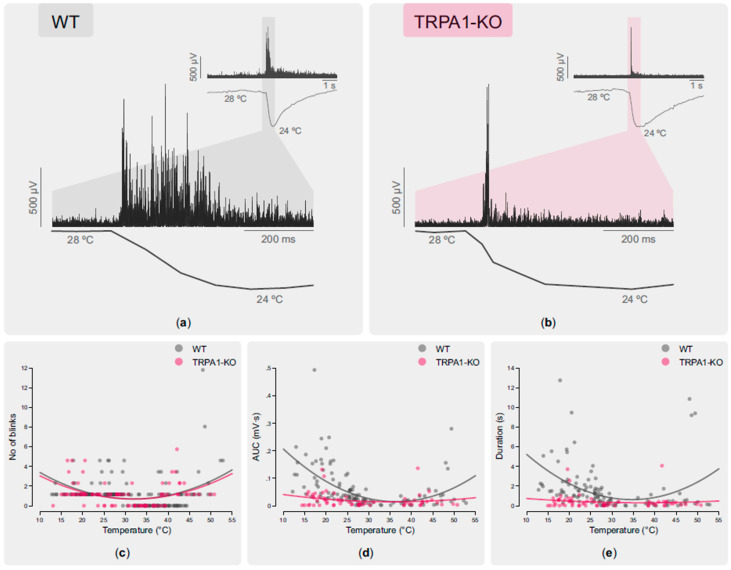
Sample recordings of OOemg signals evoked as reflex response to the instillation of a 5 μL saline drop onto the ocular surface of a WT (**a**) and a TRPA1-KO (**b**) mouse; lower traces represent the ocular surface temperature, in both cases showing the temperature decrease induced by cooling drops. The number of blinks evoked by each stimulus applied onto the ocular surface (**c**), the area under the curve (AUC) (**d**), and the duration (**e**) of the OOemg signals are shown. Each dot represents a single stimulus. The continuous lines are second order regression lines.

Application onto the ocular surface of a 5 µL drop of saline solution at different temperatures (13–43 °C) evoked a reflex blinking response in anesthetized mice. Both the OOemg signal and the ocular surface temperature were recorded continuously to analyze the changes induced by each stimulus (Figure 4a,b) (see Methods for details).

The number of blinks evoked by the stimulus was proportional to its intensity, both for cooling and heating stimulation (Table 3; Figure 4c), with no significant differences between the WT and TRPA1-KO groups. For cooling stimulation, the amplitude (area under the curve, AUC) and duration of the OOemg signal strongly correlated with the intensity of cooling in the WT mice (Table 3; Figure 4d,e). Only a weak correlation between the OOemg AUC and the cold stimulus intensity was found in the TRPA1-KO mice (Table 3; Figure 4d,e). For heating stimulation, the intensity of the stimulus did not correlate with the AUC and duration of the OOemg signals, neither for the WT nor for the TRPA1-KO mice (Figure 4d,e). These data suggest that TRPA1 is involved in cold- but not in heat-evoked reflex blink in mice.

### 2.3. Effects of Aging in TRPA1-Dependent Thermosensitivity

The cold thermoreceptor activity in aged (18-month-old) WT and TRPA1-KO mice was recorded and compared to determine the changes associated with aging in both groups.

#### 2.3.1. Background and Stimulus-Evoked Activity of Cold Thermoreceptors

The background activity and response to cooling stimulation (from 34 °C to 15 °C) of the corneal cold thermoreceptors recorded in aged WT and aged TRPA1-KO mice eyes had similar values to those in young WT and young TRPA1-KO mice (Table 1, Figure 1). Furthermore, the proportion of the LB-LT cold thermoreceptors was similar in the aged TRPA1-KO and in the young TRPA1-KO mice (45.5% aged TRPA1-KO vs 55.6% young TRPA1-KO, *p* = 1.0, z-test; Figure 2, pink triangles and circles, respectively). When comparing the activity of the cold thermoreceptors to warming (from 15 °C to 34 °C), the aged WT mice displayed both a higher temperature threshold and a longer return time values than the young WT (Table 1, Figure 1). Remarkably, the corneal cold thermoreceptors of young TRPA1-KO mice showed a warming response similar to the aged TRPA1-KO and WT mice, taking more time to recover the impulse activity than the young WT (Table 1, Figure 1). Similarly, when comparing the response to heating (from 34 °C to 45 °C), the cold nerve terminals of the young and aged TRPA1-KO and the aged WT mice silenced at slightly higher temperatures and had a higher heating threshold than young WT, although the differences did not reach statistical significance (Table 1). Altogether, these results further support the involvement of TRPA1 in the response to warming, and indicate that in the absence of TRPA1, cold thermoreceptors from young mice behave in a similar way to those of aged mice.

#### 2.3.2. Shape of the Nerve Terminal Impulse

As in recordings in young mice eyes, the shape of the NTIs of the corneal cold thermoreceptors was biphasic in aged TRPA1-KO animals. The NTIs of the aged WT and TRPA1-KO cold nerve terminals showed a slower maximum rate of voltage change during the upstroke (+dV/dt) and a longer depolarization time (T1) than young animals (Table 2), although the differences were significant only when comparing the young and aged WT (*p* < 0.050). These results suggest that the changes in the NTI shape developed with age are attributable to other causes rather than the absence or presence of TRPA1.

#### 2.3.3. Basal Tearing Volume

The basal tearing volume was significantly higher in the aged TRPA1-KO animals compared to the young TRPA1-KO mice (1.5 ± 0.4 mm vs. 4.11 ± 0.42 mm, n = 8 and 17 for young and aged, respectively; *p* < 0.001, *t*-test). Similar differences were observed between the young and aged WT mice (1.3 ± 0.3 mm vs. 3.4 ± 0.8 mm, n = 10 and 10 respectively; *p* = 0.021, Mann–Whitney test). The data support the idea that TRPA1 is not involved in basal tearing or in its change with age.

## 3. Discussion

The present data show that the TRPA1 channel is not necessary to determine the background firing activity and the shape of nerve terminal impulses of corneal cold thermoreceptors at basal temperature. Moreover, TRPA1 is neither essential for the cold-evoked activity nor the heat-evoked activity of corneal cold thermoreceptors. In the same way, TRPA1-dependent activity seems not to be fundamental for the maintenance of basal tearing rate or the reflex blink response evoked by thermal stimulation of the ocular surface. However, in mice where TRPA1 was knocked out, most of the cold thermoreceptors presented an altered background activity, thus being classified as a low background–low threshold (LB-LT) cold thermoreceptor [27]. Their activity in response to heating was also altered, resembling that found in aged WT mice. Moreover, when TRPA1 was not expressed, the magnitude and duration of the OOemg signal evoked by ocular surface temperature changes did not correlate with the magnitude of the thermal change. The present electrophysiological data suggest that in WT animals, where TRPA1 is present, there is a decrease with age in their level of expression and/or activity. Although TRPA1 is not essential for corneal cold thermoreceptors’ thermosensitivity, this channel seems to be important for the detection of warming and to encode the ocular surface temperature changes, thus contributing to defining the magnitude of the thermally-evoked reflex blink.

The corneal cold thermoreceptors fire nerve impulses spontaneously (background activity) at ocular surface basal temperature (34–35 °C), often in bursts [16,24,29]. This background activity was not significantly modified in young TRPA1-KO animals compared with young WT. Also, aged TRPA1-KO and WT mice had a background activity at 34 °C similar to young mice. This result is consistent with a previous report [40] showing that 72.5% of the corneal nerve terminals recorded from 24-month-old WT mice responded as those recorded in young animals. Altogether, the present results indicate that TRPA1 has a minor role in determining cold thermoreceptor background activity at basal temperature, reinforcing that TRPM8 is the primarily involved channel [31].

When recorded extracellularly, as with the focal recording technique used in this work, the changes in the recorded potential are proportional to the net membrane current. Under this assumption, the shape of the recorded NTI represents the first derivative of the membrane voltage change, which is predominantly outward during the spike depolarization (thus generating a positive peak in the NTI recording) and inward during the spike repolarization (thus recorded as a negative peak) [44,45]. The current generated through K^+^ channels is on the basis of the variations over time generating the recorded NTIs [42]. Some types of K^+^ channels such as the sodium-activated K^+^ channel Slack (KNa1.1 or Slo2.2) play a key role in shaping the neuronal electrical properties [46,47,48] and in modulating the TRPA1-mediated nociception by interacting functionally with this channel [43]. The pharmacological inhibition of TRPA1 by A-967079 decreases the total outward potassium current (Ik) in sensory neurons and in a HEK cell line transfected with TRPA1 and Slack [43]. In that regard, it could be expected that the complete absence of TRPA1 may affect K^+^ currents, thus modifying the shape of the NTIs. However, we found that the genetic ablation of the TRPA1 channels did not produce changes in the NTI shape regardless of age. There may be compensatory mechanisms for the absence of the TRPA1 channel (which constitutes an important difference with respect to its pharmacological blockade), thus explaining why the absence of TRPA1 expression did not affect the NTI shape. On the other hand, the differences found in the young and aged WT animals suggest the inactivation or downregulation of K^+^ channels with aging, as the changes observed in different NTI parameters were similar to those produced by the K^+^ channel blockers [45].

By analyzing the cold thermoreceptors’ nerve activity during the cooling ramp, we did not find significant differences between the WT and TRPA1-KO animals, regardless of the age of mice. However, as the amplitude of NTIs was reduced during cooling and completely silenced at temperatures below 27 °C in both the WT and TRPA1-KO animals [27], we cannot assign a role to TRPA1 in the response to cooling between 27 °C and 15 °C. The present results are consistent with previous studies, where no differences in cold sensitivity were observed in TRPA1-KO and WT mice [6,7,49]. On the other hand, although the similarities between the background activity and response to cooling of cold thermoreceptors recorded in WT and TRPA1-KO corneas seem to suggest that TRPA1 is not involved in defining the background firing and cold sensitivity, there are small differences when classifying them into HB-LT, LB-HT, or the intermediates LB-LT and HB-HT according to their background activity at 34 °C and cooling threshold. When TRPA1 is knocked out, there is a larger proportion of LB-LT cold thermoreceptors, similar to what happens in very old mice [40] and in injured corneas [42].

During the cooling ramp from 34 °C to 15 °C cold nerve terminals become silenced. When warming from 15 °C back to 34 °C, the activity starts reappearing when the temperature is about 30 °C [27]. In both young and aged TRPA1-KO mice, the time elapsed for the regained nerve impulse activity (as well as the temperature needed to achieve it) was significantly increased compared to the young WT. This effect was also found in aged WT. These results suggest that (1) TRPA1 is involved in the warming response after a cooling stimulus (in the range between 15 °C and 34 °C), and (2) the responsiveness of TRPA1 is affected by age. 

It has been suggested that TRPA1 contributes also to sensing noxious heating [11,12]. When heating in the non-painful range (from 34 °C to 40 °C), some cold thermoreceptors are silenced. After that, some of them start to fire NTIs when the temperature passes over 40 °C (the so called paradoxical response to heat of cold thermoreceptors) [27,32,36]. It has been reported that silencing of cold thermoreceptors when heating stimuli are applied is essential for skin warmth perception [50]. When compared to the cold thermoreceptors that were silenced by warming, the silencing temperature tended to be higher in young and aged TRPA1-KO and in aged WT than in young WT. In the paradoxical response to heat, the heating threshold values were also slightly higher in the TRPA1-KO mice, as previously reported in these animals [11]. Altogether, these results support the idea that TRPA1 contributes to detecting temperature increases, together with other thermosensitive channels present in cold sensory neurons such as TRPV1 or TRPM3 that support heating responses [12].

The changes in the expression and or activity of the TRP channels during aging has been rarely studied. Although morphological and functional changes with age of cold thermoreceptors expressing TRPM8 channel have been reported [40], so far nothing has been reported about TRPA1. The present results show that when the TRPA1 is not present, there is an increase in the proportion of cold thermoreceptor terminals classified in the intermediate group LB-LT. Moreover, these terminals present a delay in the recovery of cold thermoreceptors’ activity when warming after the cooling ramp, as well as a change in the silencing temperature when heating and the heating threshold. These alterations of the response to heat are also present in aged WT mice [40]. The absence of TRPA1 affects the function of other tissues. For instance, the lack of TRPA1 accelerates the development of age-related cardiac fibrosis, ventricular dilation, and cardiac dysfunction [51]. The results support the hypothesis that the absence of TRPA1 accelerates the changes induced by aging. It has been described that the response to both non-noxious warm and cold tend to decline gradually with age, with the ability to perceive non-noxious warm stimuli strongly reduced, while the pain sensations evoked by noxious heat seem to be preserved [38]. The present results suggest that the cold thermoreceptors of the TRPA1-KO mice behave as the cold thermoreceptors of the WT aged mice, with reduced warming sensitivity.

The basal tearing rate depends on the level of background activity of the corneal cold thermoreceptors at basal temperature [31]. In this work, we set out to establish whether TRPA1 channels also play a role in the basal tearing regulatory mechanism. No differences in basal tearing were found between the young WT and young TRPA1-KO, and the significant increase in the basal tearing observed in aged WT [40] was also present in aged TRPA1-KO. This suggests that although TRPA1 is involved in tearing under inflammatory conditions [32,36] it is not primarily involved in basal tearing under nonpathological conditions, which depends mainly on the activity of the cold-sensing channel TRPM8 [31].

The sensory inflow originated by cold thermoreceptor activity is also used by the central nervous system to control basal blinking [33,52]. Although polymodal nociceptor activity is responsible for inducing reflex blinking in healthy volunteers [30], reflex blink is evoked also by non-noxious thermal stimulation of the ocular surface. Here, we studied the contribution of TRPA1-mediated activity to the frequency and characteristics of reflex blinking evoked by thermal stimuli applied on the ocular surface. While the number of blinks evoked by a defined thermal stimulus was similar in the WT and TRPA1-KO animals and proportional to the ocular surface temperature change, the characteristics of blinks (AUC and duration of the OOemg signal) were different. Previous work performed in HEK cells transfected with human (hTRPA1) and mouse (mTRPA1) TRPA1 [9] and in planar lipid bilayers with reconstituted hTRPA1 [10,53] indicated that both hTRPA1 and mTRPA1 are dual heat and cold thermoreceptors, as the channel opens with a U-shape thermosensitivity. Our results show that in WT, the OOemg parameters tended to increase with the temperature change, resembling the U-shape of the TRPA1 channel opening in response to thermal stimulation [10]. This correlation between the intensity of the corneal temperature change and the amplitude of the evoked blink was not present in TRPA1-KO mice. These data suggest that the activity of the corneal cold thermoreceptors encodes the ocular surface temperature change and, in turn, defines the amplitude, duration, and frequency of the reflex blink response. The data obtained in the TRPA1-KO mice suggest that the cold thermoreceptor activity mediated by TRPA1 contributes to encoding ocular surface temperature changes, mainly innocuous warming. However, the contribution of the activity of other corneal sensory nerve terminals (such as polymodal nociceptors or thermoreceptors from the bulbar or palpebral conjunctiva) to detect the temperature changes produced by cooling or heating cannot be fully ruled out. 

The present data show that the absence of TRPA1 does not abolish the cold and heat thermosensitivity of corneal cold thermoreceptors under nonpathological conditions, which explains normal basal tearing of TRPA1-KO mice. However, TRPA1 is involved in ocular pain sensation during inflammation [32,36,54], being upregulated by the proinflammatory molecules found in tears of dry-eye disease patients [37]. It is reasonable to think that under corneal inflammation or injury, the involvement of TRPA1 in corneal thermosensitivity could be increased, although this still needs to be deeply studied.

In summary, TRPA1 contributes to corneal thermosensitivity under nonpathological conditions, having a role in the sensory inflow to the central nervous system in response to both cooling and heating of the ocular surface. TRPA1 is especially relevant for the generation of the nerve impulse activity during the non-noxious warming of cold thermoreceptors. Although the absence of TRPA1 does not eliminate the response of cold thermoreceptors to cooling and noxious heating stimulation, its presence and the TRPA1-dependent activity of corneal cold thermoreceptors contribute to codifying the intensity of the temperature change, evoking blink reflexes proportional to thermal changes.

## 4. Methods

### 4.1. Animals

Young (5 months) and aged (18 months) wildtype C57BL/6 (WT) and genetically modified homozygous TRPA1^−/−^ knockout mice with C57BL/6 background (TRPA1-KO) were used. Male and female animals were included. All the animals were bred and provided by the animal facility of our university. All the experiments were conducted in accordance with the institutional animal care guidelines and according to the Spanish Biomedical Research Act and the European Union Directive 2010/63/EU on the protection of animals used for scientific purposes. All experimental procedures were carried out according to the Spanish Royal Decree 53/2013 and followed a protocol approved by the Committee of Ethics and Integrity in Research (CEIR) of the Universidad Miguel Hernández de Elche, and the Generalitat Valenciana.

### 4.2. Electrophysiological Recording

**Figure 5:**
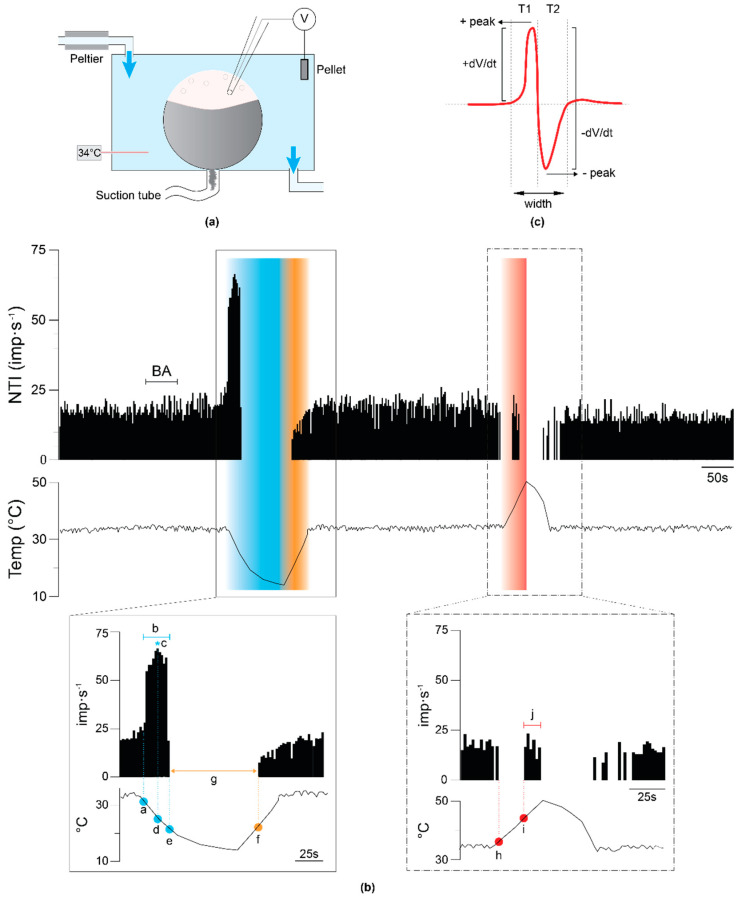
Electrophysiological recording setup and analysis. (**a**) Schematic diagram of the chamber showing the different points over the corneal surface at which the recording electrode is placed during the experiment. (**b**) Example of the NTI activity of a corneal nerve terminal recorded in the cornea of a wildtype mouse during the complete experimental protocol, showing the background activity (BA) and the response to cooling and heating ramps. Upper trace: Histogram of the firing frequency in impulses per second. Lower trace: Recording of the temperature of the perfusion solution in °C. Blue and red bands have been added to help visualizing the terminal discharge rate during the cooling and heating ramps, respectively. The insets show the parameters measured to quantify the responses to the cooling and heating stimuli: (a) cooling threshold; (b) cooling response; (c) peak frequency, that is, the maximum frequency per second during the cooling ramp indicated by the blue asterisk; (d) temperature at the peak frequency; (e) silencing temperature when cooling from 34 °C to 15 °C; (f) temperature at which NTI activity returns when heating from 15 °C to 34 °C; (g) return time; (h) silencing temperature during the heating ramp from 34 °C to 45 °C; (i) heating threshold; (j) heating response. (**c**) Parameters measured to define the NTI shape. The positive and negative peak amplitude (+peak, –peak), the maximum rate of voltage change during the initial upstroke and the downstroke (+dV/dt max, −dV/dt max), the NTI duration (width), and the depolarization and repolarization time (T1, T2) were measured. The ratio between the maximum rate of voltage during the initial upstroke and during the downstroke was then calculated.

Recordings of single corneal nerve terminals were performed ex vivo as described in previous studies [26,31,55]. The mice were sacrificed with an overdose of sodium pentobarbitone (Dolethal^®^, Ventoquinol, France) injected intraperitoneally. Then, a 10/0 silk thread was used to sew a point at the temporal conjunctiva, near the limbus, and both eyes were enucleated along with a short length of the optic nerve and surrounding tissues. The excised eyes were then pinned to the bottom of a silicone-coated (Sylgard 154^®^, Dow Corning, Freeland, MI, USA) chamber and secured in place by continuous suction (Figure 5a). The eye was continuously superfused with physiological saline solution of the following composition (in mM): NaCl (128), KCl (5), NaH_2_PO_4_ (1), NaHCO_3_ (26), CaCl_2_ (2.4), MgCl_2_ (1.3), and glucose (10). The solution was gassed with carbogen (5% CO_2_ and 95% O_2_) to pH 7.4 and maintained at the desired temperature (basal temperature ~34 °C) with a homemade Peltier device.

A borosilicate glass micropipette electrode with a tip diameter of about 50 µm filled with saline solution was gently placed in contact with the corneal surface using a micromanipulator. Light suction was then applied through the pipette to produce a high-resistance seal with the corneal surface, allowing the recording of the nerve impulses generated at the single nerve terminals located beneath electrode tip (Figure 5a). The electrical signals were recorded with respect to an Ag/AgCl reference electrode placed inside the recording chamber (Figure 5a).

The nerve terminal impulses (NTIs) were amplified with an AC amplifier (Neurolog NL104, Digitimer, Welwyn, UK), filtered (high pass 1 Hz, low pass 5 kHz; filter module NL124, Digitimer, Welwyn, UK), and stored at 25 kHz sampling into a computer using a CED micro-1401 interface and Spike2 v.8.02 software (both from Cambridge Electronic Design, Cambridge, UK). Only recordings containing NTIs originating from a single nerve terminal were analyzed.

Thermal stimulation was conducted by decreasing or increasing the basal temperature of the perfusion solution from 34 °C (down to 15 °C or up to 45 °C) using the Peltier device. 

#### 4.2.1. Experimental Protocol

The recording pipette was placed at defined points over the corneal surface. The stitch performed in the temporal conjunctiva was used as a reference to facilitate the mapping of the recorded unit on the corneal surface, helping to define the location of the different recorded terminals. The eye was rotated as necessary to explore the whole corneal surface.

After application of the pipette to the corneal surface, the appearance of spontaneous or stimulus-evoked NTI activity at the recording site was used to ascertain success in detecting an active sensory nerve terminal. Responses to cold stimuli were assessed. If no spontaneous or stimulus-evoked activity was obtained, the electrode was moved to the next recording point.

First, cold stimulation was performed by decreasing the temperature of the perfusion solution from 34 °C to 15 °C at a ~0.25 °C/s rate. When the peak temperature decrease was attained, warming was applied to return to basal temperature. After a resting period of 5 min, a heating ramp from 34 °C to 45 °C was carried out at ~0.25 °C/s rate (Figure 5b). 

#### 4.2.2. Analysis of the Electrophysiological Recordings

Offline analysis of the electrophysiological recordings was performed using Spike2 v.8.02 software. The NTIs detected during the first acquisition were filtered using a threshold-based criterion to distinguish them from noise. Although changes in the amplitude of NTIs were observed along the recording time, particularly associated with the decrease in the temperature [25], the software analysis used was able to group in the same cluster all the spikes with amplitudes and shapes corresponding to a single unit.

##### 4.2.2.1. Background and Stimulus-Evoked NTI Activity

Different parameters of the NTI activity were calculated to define the characteristics of the spontaneous activity and the cold- and heat-evoked activity of the cold thermoreceptor terminals (Figure 5b, insets). First, the *Background activity* (BA), defined as the mean basal ongoing frequency in impulses per second (imp.s^−1^), at basal temperature (33.5 ± 0.16 °C) was measured during a 30 s period before any intended stimulation was calculated. Also, the following parameters were calculated: (a) *Cooling threshold*, the temperature value (in °C) during the cooling ramp at which the NTI frequency increased to a value 25% greater than the background NTI activity. (b) *Cooling response*, the mean discharge rate (in imp.s^−1^) in response to the cooling ramp. (c) *Peak frequency* (PF), the maximal firing frequency reached during the cooling ramp (in imp.s^−1^). (d) *Temperature to reach the peak frequency*, the temperature (in °C) at which PF occurred. (e) *Silencing during temperature cooling*, the temperature (in °C) at which the impulse discharge silenced during the cooling ramp. (f) *Return temperature*, the temperature (in °C) at which the impulse discharge started when recovering the basal temperature after the cooling ramp. (g) *Return time*, the time (in s) elapsed since the NTI was silenced during the cooling ramp, and it started over when heating to the basal temperature after the cooling ramp. (h) *Silencing during temperature heating*, the temperature (in °C) at which the impulse discharge was silenced during the heating ramp. (i) *Heating threshold*, the temperature (in °C) required to evoke firing in response to a heating ramp. (j) *Response to heat*, the mean discharge rate (imp.s^−1^) in response to the heating ramp.

Some of the measured parameters allow classifying cold thermoreceptors into different functional types. When the background activity at 34 °C was equal or over 1.5 imp.s^−1^, the cold thermoreceptors were considered high background (HB); otherwise, they were considered low background (LB). When the cooling threshold during the cooling ramp from 34 °C to 15 °C was equal to or below 30.5 °C, they were considered high threshold (HT); otherwise, they were considered low threshold (LT). Based on their background activity at basal temperature and their cooling threshold, corneal cold thermoreceptors are classified as high background–low threshold (HB-LT) and low background–high threshold cold thermoreceptors (LB-HT) [16,27], with intermediate behaviors, that is, high background–high threshold (HB-HT) and low background–low threshold (LB-LT), present under certain pathological condition and in aged animals [40,41].

##### 4.2.2.2. Shape of the NTIs

We also analyzed the shape of the NTIs recorded from cold thermoreceptors during their background activity at basal temperature. Based on a previous study [56], the parameters measured to define the shape of the nerve terminal impulse were (Figure 4c): the *positive peak amplitude* (+peak, mV), the *negative peak amplitude* (–peak, mV), the *maximum rate of voltage change during the initial upstroke* (+dV/dt max, mV/ms), the *maximum rate of voltage change during the downstroke of the NTI* (−dV/dt max, mV/ms), the *ratio between +dV/dt max and −dV/dt max* (Ratio), the *total duration of the NTI* (Width, ms), the *depolarization time* (T1, ms), and the *repolarization time* (T2, ms).

### 4.3. Measurement of the Basal Tearing Volume

The tearing rate was measured in both eyes in deeply anesthetized mice using commercial phenol red threads (Zone-Quick, Menicon, Nagoya, Japan) placed between the lower lid and the bulbar conjunctiva in the temporal side of the eye for 30 s. These threads are yellow when dry and change to red when wetted in contact with tears due to the pH change; thus, the length of the red-colored thread reflects the amount of tears. The tear volume is expressed as the entire length of the red portion of the thread in mm, measured with a calibrated scale under a stereomicroscope. Both eyes for each animal were measured sequentially, and the values obtained were pooled.

### 4.4. Orbicularis Oculi Muscle Electromyography (OOemg)

#### 4.4.1. Electrode Implantation

Animals were anesthetized with 3% isoflurane (3% for induction, 1–1.5% for maintenance; IsoFlo^®^, ESTEVE, Barcelona, Spain) and placed on a surgical table. Body temperature was maintained with a feedback thermal blanket (Harvard Apparatus, Holliston, MA, USA). Vital functions such as heart rate and arterial O_2_ saturation were monitored continuously using a pulse oximeter designed for mice and rats (Mouse-Ox^®^ Pulse Oximeter, Ugo Basile, Comerio, Italy). Implantation of electrodes to record the orbicularis oculi electromyographic signal was performed following a procedure described previously [57]. Briefly, custom-made electrodes were built with Teflon-insulated stainless-steel wire (50 µm in diameter, A-M Systems, Sequim, WA, USA). A pair of electrodes was implanted within the fibers of the orbicularis oculi muscle and connected to a 6-pin socket, which was attached with acrylic dental resin (Duralay^®^, Reliance, IL, USA) to the skull surface. Analgesic treatment was delivered (Buprecare 0.3 mg/mL, Haupt Pharma Livron S.A.S, Livron-sur-Drôme France), and the animals were allowed to recover in individual cages for 10 days prior to the EMG recording procedure.

#### 4.4.2. OOemg Recording

The animals were anesthetized with an intraperitoneal injection of urethane (1.5 g/Kg) and immobilized in a stereotaxic frame. The implanted electrodes were connected to an amplifier (1000×; DAM80, WPI Sarasota, FL, USA), and the signal was filtered (pass band 300–3000 Hz; DAM80), digitized (25 kHz sampling rate; Micro 3-1401, CED, Cambridge, UK), and stored for offline analysis using specific software (Spike2 v8.02, CED, Cambridge, UK). Several 5 µL drops of saline solution at different temperatures (ranged from 13 °C to 43 °C) were instilled on the eye at 30 s^−1^ min intervals. Each drop induced a change in the ocular surface temperature that was measured with a high-speed ultrafine flexible thermal probe (IT-24P, Physitemp Bat-12, Clifton, NJ, USA) placed in contact with the cornea to allow the continuous recording of the temperature. For ethical reasons, the electrode implantation and OOemg recording procedures were performed only in young animals, based on the low success rate of the procedures in aged mice.

#### 4.4.3. OOemg Analysis

The number of blinks evoked by each thermal stimulus applied on the cornea was counted. Also, two parameters of the OOemg signal evoked by each temperature stimulus were analyzed using Spike2: *duration* (in s) of the evoked EMG response and *area under the curve* (AUC; in mV·s) of the evoked EMG response.

### 4.5. Statistical Analysis

Data were collected and processed for statistical analysis using SigmaStat software (SigmaStat v3.5; Systat Software Inc., Point Richmond, CA, USA). The values are expressed as mean ± SEM, with n denoting the number of terminals, eyes, or EMG recordings as indicated.

Differences between groups were compared with One Way ANOVA or ANOVA on Ranks depending on the normality of the data and their suitable post hoc as indicated in the text, with the young WT group used as the default control group for all comparisons. The correlations were calculated using the Pearson correlation test. *p* = 0.05 or below was considered significant.

## Figures and Tables

**Table 1:** Characteristics of the background activity and the response to thermal stimulation of corneal cold nerve terminals recorded in young (5-month-old) and aged (18-month-old) wildtype and TRPA1-KO mice.

	Young Mice		Aged Mice	
	WT n = 14	TRPA1-KO n = 9	WT n = 11	TRPA1-KO n = 11
**Basal temperature of 34 °C**	4.1 ± 0.7	3.4 ± 1.3	7.7 ± 1.2	4.5 ± 2.2
Background activity (mean discharge rate; imp.s^−1^)				
**Cooling from 34 °C to 15 °C**	31.6 ± 0.3	31.8 ± 0.4	32.2 ± 0.5	32.5 ± 0.5
Cooling threshold (°C)				
Cooling response (mean discharge rate; imp.s^−1^)	14.9 ± 2.5	19.8 ± 3.2	16.9 ± 2	13.0 ± 3.8
Peak frequency (PF; imp.s^−1^)	28.6 ± 4.9	40.3 ± 5.1	30.5 ± 3.3	25.8 ± 6.6
Temperature at PF (°C)	29 ± 0.9	29.7 ± 0.9	31.2 ± 0.8	30.5 ± 1.1
Silencing temperature during the cooling ramp (°C)	26.9 ± 0.9	27.7 ± 1.2	30 ± 1	28.6 ± 1.3
**Warming from 15 °C to 34 °C**	28.9 ± 0.7	33.2 ± 0.6 *	32.4 ± 0.6 *	31.9 ± 0.5 *
Return temperature (temperature to recover impulse activity; °C)				
Return time (time to recover impulse activity; s)	74.2 ± 8.6	164.5 ± 23 *	167.5 ± 16.5 *	131.3 ± 15.2 *
**Heating from 34 °C to 45 °C**	4/9	6/9	4/11	3/11
Terminals fully silenced during the heating ramp (silenced terminals/n)				
Silencing temperature during the heating ramp (°C)	35.4 ± 0.8	38.3 ± 1.5	39.9 ± 1.8	38.6 ± 2.4
Terminals responding to heat (responding terminals/n)	9/12	6/9	3/11	6/11
Heating threshold (°C)	41.1 ± 1.3	43.7 ± 0.8	44.7 ± 1.0	43.2 ± 1.9
Response to heat (mean discharge rate; imp.s^−1^)	11.5 ± 4.6	16.2 ± 6.3	10.2 ± 5.1	12.1 ± 3.4

n = number of recorded cold nerve terminals. Data are mean ± SEM. * *p* < 0.05, ANOVA One Way with Dunn’s or the Holm–Sidak method, differences from young WT mice. To make the table more readable, columns showing data from TRPA1-KO mice have been colored.

**Table 2:** Parameters defining the shape of the nerve terminal impulses (NTIs) of the corneal cold thermoreceptors at basal temperature in young (5-month-old) and aged (18-month-old) wildtype and TRPA1-KO mice.

	Young Mice		Aged Mice	
	WT n = 13	TRPA1-KO n = 9	WT n = 7	TRPA1-KO n = 9
+dV/dt max (mV/ms)	3.02 ± 0.14	3.15 ± 0.13	2.46 ± 0.25	2.79 ± 0.28
−dV/dt max (mV/ms)	−3.25 ± 0.14	−3.32 ± 0.19	−2.83 ± 0.31	−3.10 ± 0.19
Ratio	−0.95 ± 0.06	−0.97 ± 0.06	−0.89 ± 0.06	−0.93 ± 0.09
Width (ms)	1.5 ± 0.09	1.4 ± 0.07	1.8 ± 0.17	1.5 ± 0.07
T1 (ms)	0.5 ± 0.02	0.5 ± 0.02	0.7 ± 0.06 *	0.6 ± 0.05
T2 (ms)	1.0 ± 0.09	0.9 ± 0.06	1.2 ± 0.1	0.8 ± 0.05

n = number of recorded cold nerve terminals. * *p* < 0.05, ANOVA One Way with Dunn’s or the Holm–Sidak method, differences from young WT. To make the table more readable, columns showing data from TRPA1-KO mice have been colored.

**Table 3:** Pearson correlation coefficient (r) between the amplitude of the ocular surface temperature change induced by thermal stimulation (cooling and heating), and the number of reflex blinks and characteristics (area under the curve, AUC, and duration) of the OOemg signal recorded in young WT and TRPA1-KO mice.

	WT	TRPA1-KO
**Cooling**		
No. of blinks	r =−0.206 ***p* = 0.047** n = 94	r = −0.484 ***p* = 0.00005** n = 64
OOemg AUC	r = −0.556 ***p* = 0.00000003** n= 85	r = −0.339 ***p* = 0.023** n = 45
OOemg duration	r = −0.457 ***p* = 0.00001** n = 85	r = −0.243 *p* = 0.108 n = 45
**Heating**		
No. of blinks	r = 0.517 ***p* = 0.000002** n = 75	r = 0.423 ***p* = 0.014** n = 33
OOemg AUC	r = 0.366 *p* = 0.061 n = 26	r = −0.08 *p* = 0.747 n = 20
OOemg duration	r = 0.324 *p* = 0.106 n = 27	r = −0.09 *p* = 0.706 n = 20

n = number of stimuli applied to the ocular surface. Columns showing data from TRPA1-KO mice have been colored.

## Data Availability

The original contributions presented in the study are included in the article. Further inquiries can be directed to the corresponding author.
